# Bisphenol A Disrupts Transcription and Decreases Viability in Aging Vascular Endothelial Cells

**DOI:** 10.3390/ijms150915791

**Published:** 2014-09-09

**Authors:** Edna Ribeiro-Varandas, H. Sofia Pereira, Sara Monteiro, Elsa Neves, Luísa Brito, Ricardo Boavida Ferreira, Wanda Viegas, Margarida Delgado

**Affiliations:** 1 Centro de Botânica Aplicada à Agricultura (CBAA), Instituto Superior de Agronomia, Universidade de Lisboa, Tapada da Ajuda, 1349-017 Lisboa, Portugal; E-Mails: ednavarandas@isa.ulisboa.pt (E.R.-V.); sofiapereira@isa.ulisboa.pt (H.S.P.); smonteiro@isa.ulisboa.pt (S.M.); elsa.neves@standre.ipiaget.pt (E.N.); lbrito@isa.ulisboa.pt (L.B.); rbferreira@isa.ulisboa.pt (R.B.F.); wandaviegas@isa.ulisboa.pt (W.V.); 2 Escola Superior de Tecnologia e Gestão Jean Piaget do Litoral Alentejano (ESTGJPLA), Instituto Piaget, Campus Académico de Santo André, Ap. 38, 7500-999 Vila Nova de Santo André, Portugal; 3 Instituto de Tecnologia Química e Biológica (ITQB), Universidade Nova de Lisboa (UNL), Av. da República, 2780-157 Oeiras, Portugal

**Keywords:** bisphenol A (BPA), HT29, HUVEC, LINE 1 transcription, cellular aging

## Abstract

Bisphenol A (BPA) is a widely utilized endocrine disruptor capable of mimicking endogenous hormones, employed in the manufacture of numerous consumer products, thereby interfering with physiological cellular functions. Recent research has shown that BPA alters epigenetic cellular mechanisms in mammals and may be correlated to enhanced cellular senescence. Here, the effects of BPA at 10 ng/mL and 1 µg/mL, concentrations found in human samples, were analyzed on HT29 human colon adenocarcinona cell line and Human Umbilical Vein Endothelial Cells (HUVEC). Quantitative Real-Time Polymerase Chain Reaction (qRT-PCR) transcriptional analysis of the Long Interspersed Element-1 (LINE-1) retroelement showed that BPA induces global transcription deregulation in both cell lines, although with more pronounced effects in HUVEC cells. Whereas there was an increase in global transcription in HT29 exclusively after 24 h of exposure, this chemical had prolonged effects on HUVEC. Immunoblotting revealed that this was not accompanied by alterations in the overall content of H3K9me2 and H3K4me3 epigenetic marks. Importantly, cell viability assays and transcriptional analysis indicated that prolonged BPA exposure affects aging processes in senescent HUVEC. To our knowledge this is the first report that BPA interferes with senescence in primary vascular endothelial cells, therefore, suggesting its association to the etiology of age-related human pathologies, such as atherosclerosis.

## 1. Introduction

Bisphenol A (BPA) is an industrial chemical, employed in the manufacture of many consumer products such as polycarbonate plastics and epoxy resins. The main source of human exposure to BPA is due to leaching from containers resulting in its ingestion [1,2]. Biomonitoring studies have showed that environmental exposure results in detectable internal BPA levels in the majority of individuals analyzed, (reviewed in [3]) and particularly high levels associated with occupational exposure [4,5]. BPA is an environmental xenoestrogen capable of triggering distinct estrogen-signaling pathways with potential consequences for human health, (reviewed in [6]). Several studies show that BPA at very low concentrations alters proliferation kinetics and expression of cell cycle related genes, (e.g., [7,8]). Moreover, BPA exposure can affect DNA methylation and consequently gene expression [9]. This was first observed in mice where BPA induced hypomethylation of the intracisternal A particle (IAP) retrotransposon results in altered expression of associated genes [10]. Furthermore, genome wide analysis of DNA methylation in the developing mouse forebrain revealed that 64% of the loci analyzed had developmental stage-dependent hyper or hypo methylation alterations associated with BPA exposure [11]. Altered transcription patterns associated with modifications in DNA methylation have also been reported for several genes in human cells exposed to BPA [12]. Although there is substantial evidence that BPA affects DNA methylation, the effects of this chemical on histone modifications remain largely unknown [9]. One report revealed that increased expression of the histone methyltransferase enhancer of Zeste Homolog 2 (*EZH2*) is induced by BPA with consequent increase in the overall level of histone H3 trimethylation at lysine 27 (H3K27me3), both in human breast cancer cells and in mouse mammary glands [13]. Recently, we have also shown that BPA alters intranucleolar distribution of two distinct H3 post-translation modifications, H3 trimethylation at lysine 4 (H3K4me3) and H3 dimethylation at lysine 9 (H3K9me2), in primary Human Umbilical Vein Endothelial Cells (HUVEC), although without alteration in the global level of these two epigenetic marks [14].

Numerous studies have established a close relationship between epigenetics and aging. Epigenetic modifications have been highly correlated with age-related pathologies, such as cancer, neurodegenerative and cardiovascular disorders, as well as physiological processes of aging itself, [15,16]. Cellular aging is characterized by continuous physiological alterations and loss of replicative capacity. In primary cell culture, after a number of population doublings in cell cultures, actively dividing cell numbers decrease as cells enter replicative senescence. Senescence can also be induced by exposure to subcytotoxic doses of stressful agents, such as oxidants or ethanol [17,18]. Accordingly, it has been recently shown that BPA may have an important role in enhanced cellular senescence in normal human mammary epithelial cells (HMEC), attributable to deregulation of cell cycle regulatory genes [19]. A relevant fact is that high urinary BPA concentrations have been correlated with pathogenesis of age-related diseases, such as coronary and carotid atherosclerosis [20,21,22], a form of accelerated arterial senescence [23]. However, despite the extensive research on BPA effects in human cancer cells, effects of continuous contact on vascular primary cells are mostly unknown.

In this work, we utilized HUVEC and HT29 human colon adenocarcinona cell line to compare BPA effects on primary cells of vascular tissue and cancer cells from digestive tract tissue. The Long Interspersed Element-1 (LINE-1 or L1) is a highly abundant retroelement distributed throughout the human genome. To access BPA effects on HT29 and HUVEC cells global transcription, we evaluated the expression of two distinct regions of LINE-1 [24]. In addition, BPA effects on cellular viability and expression of senescence associated genes were analyzed on aging HUVEC cells.

## 2. Results and Discussion

### 2.1. Bisphenol A (BPA) Alters Global Transcription in HT29 Human Colon Adenocarcinona Cell Line and Human Umbilical Vein Endothelial Cells (HUVEC)

Considering that the main route of BPA exposure in humans occurs via ingestion followed by entrance into blood circulation, HT29 cell line and HUVEC were utilized as representatives of digestive tract and vascular tissues, respectively. BPA is extensively metabolized leading to biological inactivation, however, low levels of free BPA, the bioactive form, are found in human tissues and fluids [3]. Two distinct physiological relevant concentrations of free BPA were assayed, 10 ng/mL within the range found in human samples due to environmental exposure [3,21] and 1 μg/mL associated to occupational exposure [4,5]. BPA is known to affect the transcriptional expression of several genes involved in major cellular processes in different cell lines [25,26,27,28]. In order to assess the BPA effects on general transcription, the relative transcription of two sequences from the 5' and 3' regions of LINE-1 was evaluated. LINE-1 is a ~6 kb non-site-specific autonomous, non-Long Terminal Repeat retrotransposon that comprises approximately 17% of the human genome [29] with more than 2500 L1 copies located in over 1400 genes [24].

**Figure 1:**
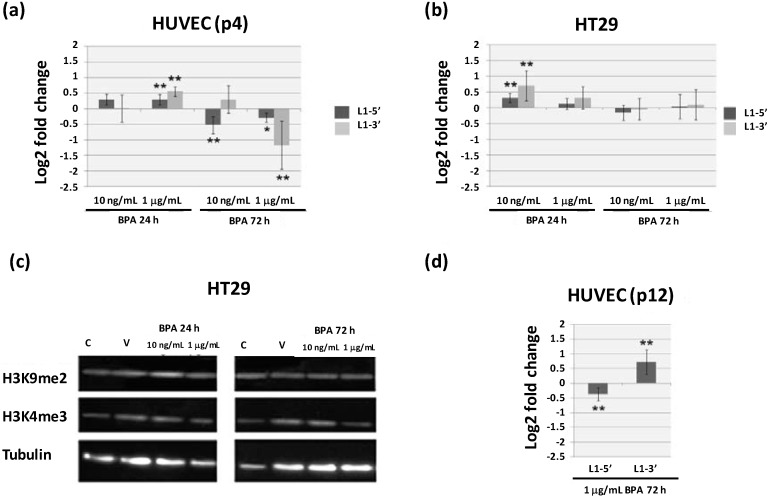
Analysis of Bisphenol A (BPA) effects on Long Interspersed Element-1 (L1) transcription levels and H3K9me2 and H3K4me3 epigenetic marks. (**a**,**b**) L1-5' and L1-3' transcriptional analysis on young (passage 4, p4) Human Umbilical Vein Endothelial Cells (HUVEC) (**a**) and human colon adenocarcinona cell line HT29 (**b**) after 24 and 72 h exposure to 10 ng/mL or 1 µg/mL BPA; (**c**) Western blotting detection of H3 dimethylation at lysine 9 (H3K9me2) and H3 trimethylation at lysine 4 (H3K4me3) on HT29 cells after 24 and 72 h exposure to 10 ng/mL or 1 µg/mL BPA showing a single 17 kDa band identical to that observed for cells grown in control medium or in medium supplemented with vehicle, α-tubulin was used as loading control; (**d**) L1-5' and L1-3' transcription levels on aging HUVEC at passage 12 (p12) after 72 h exposure to 1 µg/mL BPA. Results in (**a**), (**b**), and (**d**) are shown as the mean log2 fold change (2^−ΔΔ*C*t^) ± standard deviation in relation to equivalent cells exposed to vehicle alone. Values significantly different from vehicle are indicated, Student’s *t* test ** *p* < 0.01 and * *p* < 0.05.

Quantitative Real-Time Polymerase Chain Reaction (qRT-PCR) was performed with two sets of L1 specific primers (L1-5' and L1-3') previously utilized for the evaluation of epigenetic gene regulatory mechanisms in cancer cells [24]. Transcriptional levels were compared between BPA and vehicle (mean log2 fold change ± standard deviation) in young HUVEC cells (passage 4, p4) as well as in the immortal cell line HT29 after 24 and 72 h treatments (Figure 1a,b). L1 transcription levels were significantly altered in HUVEC cells for both exposure times, however the early response (24 h) showed to be dose dependent with both sequences being up-regulated for the higher BPA concentration (1 µg/mL) (0.295 ± 0.165 for L1-5' and 0.55 ± 0.159 for L1-3'). Interestingly, after 72 h exposure, there was a significant decrease in the expression levels of L1-5' for 10 ng/mL BPA (−0.528 ± 0.279) and in both sequences for BPA at 1 µg/mL (−0.294 ± 0.14 for L1-5' and −1.176 ± 0.77 for L1-3') (Figure 1a). These results reveal that BPA affects 5' and 3' L1 sequences differentially in a dose dependent manner, particularly for the longer exposure time (72 h). Contrary to HUVEC, in HT29 cells L1 transcription was significantly increased exclusively after 24 h BPA exposure, at the lower BPA concentration assayed (0.315 ± 0.144 in L1-5' and 0.702 ± 0.479 in L1-3' for 10 ng/mL BPA). There were no effects in L1 transcription on HT29 cells exposed to BPA for 72 h (Figure 1b), suggesting that the HT29 response to BPA is lost with prolonged exposure.

Furthermore, there is evidence that BPA exposure affects DNA methylation of various genes [12], as well as retroelement sequences [10]. However, information regarding BPA effects on histone modifications is almost inexistent [9]. Here, we analyzed BPA effects on global content of two H3 histone modifications (H3K4me3 and H3K9me2) in HT29 cells. H3K4me3 and H3K9me2 are generally associated to gene activation and silencing, respectively [30]. Immunoblotting of total protein extracts utilizing antibodies specific for each H3 modification revealed immunoreactive protein bands with the expected size of approximately 17 kDa (Figure 1c). No differences in the global content of modified histones were detected between BPA treatments and controls, as we have previously shown for HUVEC cells [14]. However, BPA induced modifications of these epigenetic marks at particular genes cannot be excluded. Previous results have shown that BPA increases expression of histone methyl transferase EZH2 resulting in increased trimethylation of H3K27 (H3K27me3), a modification associated with transcriptional repression [13]. Considering that H3K4 and H3K9 methylation is independent of EZH2 [31], our results suggest that BPA differentially affects distinct histone methylation pathways.

L1 transcription analysis revealed that young primary HUVEC are more sensitive to BPA than HT29 cancer cells, in accordance with our previous results regarding BPA micronuclei induction and transcription alteration of chromosome segregation related genes [7]. The less pronounced response to BPA in HT29 is not related with lack of expression of estrogen receptors, since both HUVEC and HT29 cells express classical estrogen receptor beta and HT29 expresses also the G protein-coupled estrogen receptor 1 (GPER) [7]. It is important to notice that BPA affinity is considerably higher for GPER than for the classical ERs and recent studies have implicated this signaling pathway in BPA cellular response [32,33]. The lower responsiveness of HT29 may therefore be related with intrinsic features of cancer cells that render them more insensitive to external signals [34]. Given the higher BPA sensitivity of HUVEC and the fact that these are primary cells that undergo senescence in culture, BPA effects on L1 transcription in HUVEC at passage 12 (p12), *i.e.*, senescent cells were also analyzed [35]. Opposite BPA effects were detected for both L1 sequences with a decrease in L1-5' (−0.377 ± 0.221) and an increase in L1-3' transcriptional levels (0.722 ± 0.423) on aging HUVEC (Figure 1d), suggesting that this chemical may induce epigenetic modifications unevenly throughout the genome. Furthermore, these results show that the effects of BPA on L1 transcription differ depending on culture time, with aging cells having a pronounced response.

### 2.2. Continuous BPA Exposure Reduces Viability in Aging HUVEC

**Figure 2:**
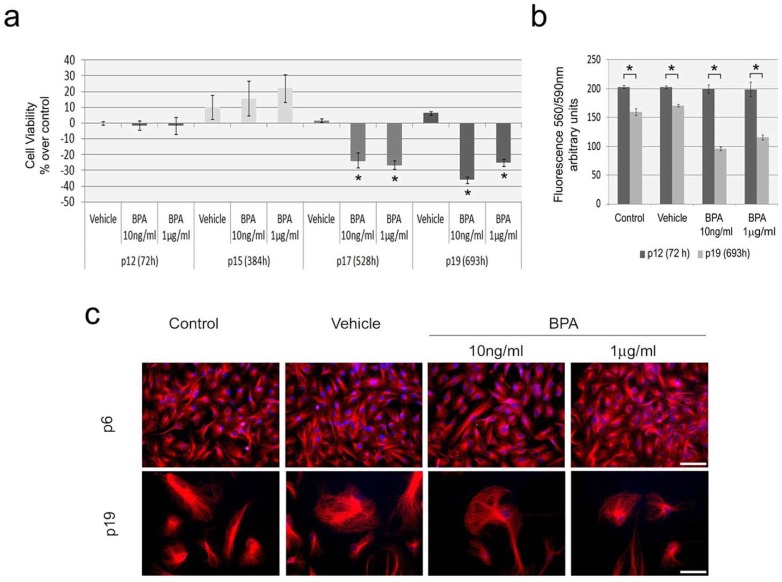
Evaluation of BPA exposure effects on cellular viability and morphology on aging HUVEC. (**a**) Variation in cell viability after 72 h exposure (passage 12, p12), 384 h (passage 15, p15), 528 h (passage 17, p17) and 693 h (passage 19, p19) of exposure to vehicle, BPA 10 ng/mL and BPA 1 μg/mL. Results are presented as percentage of variation in relation to equivalent cells maintained in standard medium (control). Student’s *t* test * *p* < 0.01 in relation to control; (**b**) Comparison of fluorescence intensity at 590 nm (excitation wavelength, 560 nm) between passage 12 and 19 for all culture conditions assayed. Results are presented as mean ± standard deviation. Student’s *t* test * *p* < 0.001. Data in (**a**) and (**b**) result from cultivation and viability procedures performed simultaneously for all growth conditions; the same seeding density was used in all passages and growth conditions; (**c**) Cell morphology analysis after in situ detection of α-tubulin (red) and DNA DAPI staining (blue) in young proliferative HUVEC (P6, **upper** panel) and aging HUVEC (P19, **lower** panels). Young proliferative HUVEC were exposed to either BPA concentration or vehicle for 72 h at passage 6; aging cells were analyzed at passage 19 after continuous exposure from passage 12. Scale bars = 50 μm.

Despite the fact that human exposure to BPA is continuous throughout life, up to now a single study on mammary epithelial cells evaluated BPA effects in cellular senescence [19]. Here, we analyzed BPA effects in cellular viability of aging HUVEC. At passage 12, HUVEC were exposed to BPA for 72 h or subjected to continuous BPA exposure for seven additional passages until passage 19 (p19). For comparison purposes, additional passages were performed regardless of the confluence level and cells were seeded at the same density for all passages/treatments. Equivalent cells were maintained for the same periods in medium supplemented with vehicle. The results are presented in relation to cells maintained under standard conditions (Figure 2a).

There were no considerable effects in cellular viability for vehicle (ethanol) in relation to control for any of the conditions assayed. More importantly, no significant variation in viability was detected after 72 h of BPA exposure at passage 12. Similarly at passage 15 (p15), corresponding to 384 h of continuous BPA exposure, no significant variation was detected between controls and treatments. Interestingly, at this passage a higher level of variation was detected independently of the growth medium, suggesting a transition phase in the senescence process. Conversely, both BPA concentrations tested induced a significant decrease in cellular viability for the longer periods, 528 h—passage 17 (p17) (−23.58% ± 4.80% and −26.60% ± 2.71%, for BPA 10 ng/mL and 1 μg/mL, respectively) and 693 h—passage 19 (−37.12% ± 1.83% and −26.26% ± 2.35%, for BPA 10 ng/mL and 1 µg/mL, respectively) (Figure 2a), indicating that concentrations found in humans as a result of environmental exposure may affect aging of vascular endothelial cells. 

A decline in cellular proliferation after prolonged culture is a common feature of primary cells associated with G0 arrest [36], and in HUVEC this is accompanied by increased spontaneous apoptosis and polyploidization [37]. Our results clearly indicate significant decreases in proliferation capacity between passages 12 and 19 (Figure 2b). This is evident as significantly lower fluorescence emission at passage 19 for all growth conditions, which indicates decreased numbers of viable cells. Relevantly, this reduction in viable cells is more aggravated in BPA treated cells, showing that prolonged BPA exposure enhances this aging related phenotype.

Cellular morphology and cytoskeleton organization was compared between non-senescent (passage 6) and senescent cells (passage 19). As aging cells cease to divide, there is the emergence of several distinctive changes in morphology, motility and mechanical strength along with cytoskeleton alterations, such as an increase in microtubules [36,38]. Immunodetection of α-tubulin clearly revealed a distinct morphology between young and aging HUVEC (Figure 2c). Senescent cells show augmented surface area, enlarged nuclear size, stellate outlines, and microtubules enrichment, as previously described [39]. Even though BPA can directly target tubulin [40] and disturb mitotic cytoskeleton on actively dividing HUVEC [7], it does not affect cytoskeleton organization on aging HUVEC. Although our cytological results did not identify alterations in cell morphology associated with BPA exposure, BPA induced effects on cell viability are supported by evident decreases in cell density at the later passage (Figure 2c).

### 2.3. Continuous BPA Exposure Induces Differential Gene Expression in Aging HUVEC

**Figure 3:**
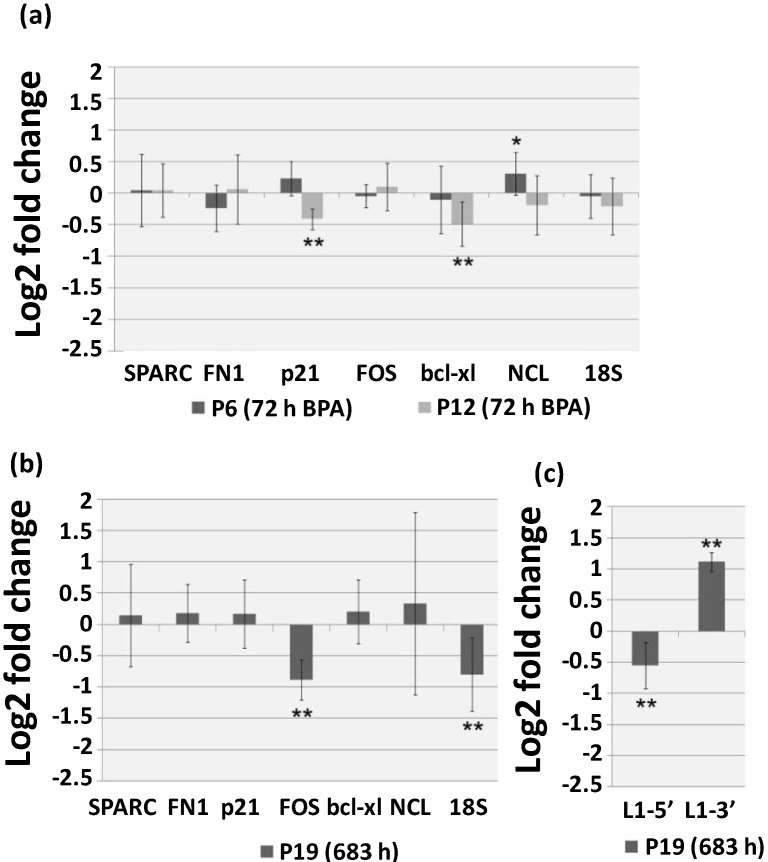
Quantitative Real-Time Polymerase Chain Reaction (qRT-PCR) evaluation of osteonectin (*SPARC*), fibronectin (*FN1*), *p21*, *FOS*, *bcl-xl*, nucleolin (*NCL*) and *18S rRNA* transcription on (**a**) young (p6) and aging (p12) HUVEC cells after 72 h of 1 µg/mL BPA exposure and (**b**) aging HUVEC (p19) after prolonged (693 h) exposure to BPA 1 µg/mL; (**c**) L1-5' and L1-3' transcription levels on aging HUVEC after prolonged exposure to BPA 1 µg/mL (p19, 693 h). Results are shown as the mean log2 fold change (2^−ΔΔ*C*t^) ± standard deviation in relation to cells from equivalent passages maintained in medium supplemented with vehicle. Student’s *t* test ** *p* < 0.01 and * *p* < 0.05.

Stress-induced senescence is associated with modifications in gene expression profiles [17,18]. In order to analyze the effects of 1 μg/mL BPA short and continuous exposure, qRT-PCR was utilized to evaluate the expression levels of seven genes on young (p6) and aging (p12 and p19) HUVEC. Four genes for which transcription is altered in replicative senescence were selected, namely two major extracellular matrix proteins coding genes, osteonectin (*SPARC*) [41], and fibronectin (*FN1*) [42], and two regulators of cell fate, *p21* and *FOS*, [37,43]. The apoptotic gene *bcl-xL* was also evaluated, since BPA exposure has been associated to alterations of transcriptional expression of apoptotic related genes in several cell lines [26,27] and age-related increase in programmed cell death was shown to occur in HUVEC cells [37]. Lastly, given that nucleolus is one of the most frequent cellular structures associated with human premature aging syndromes [44], nucleolin gene (*NCL*) and *18S rRNA* transcription levels were also analyzed. Short (72 h) exposure was evaluated in young cells (p6) and aging cells (p12) (Figure 3a) whereas continuous exposure was evaluated in aging cells maintained in the presence of BPA from passage 12 to passage 19 (p19) (Figure 3b). Results are presented in comparison to equivalent cells grown in medium supplemented with vehicle as mean log2 fold change ± standard deviation.

In young HUVEC (p6) 72 h exposure to 1 μg/mL BPA resulted, as we previously reported [14], in a slight up-regulation of *NCL* gene (0.308 ± 0.340), which encodes for nucleolin a multifunctional protein associated ribosome biogenesis as well as to several other RNA regulatory mechanisms with proliferative and survival effects [45]. Inversely, in aging cells (p12) the same BPA exposure resulted in a significant decrease of *p21* (−0.41 ± 0.166) and *bcl-xL* (−0.49 ± 0.352) transcription levels. The down-regulation of both genes suggests a lower capacity of aging cells to respond to damage, as *p21* is a CDK inhibitor with a well established role in growth arrest in response to cell injury [46,47] and *bcl-xL* encodes for a transmembrane mitochondria protein with anti-apoptotic function [48]. Seventy-two-hour BPA exposure of aging cells does not result in immediate effects on cell viability (Figure 2a) as we have also reported for young HUVEC cells [7]. However, continuous BPA exposure of aging HUVEC cells (693 h, p19) result in significant decrease in cell viability accompanied by significant down-regulation of the mRNA levels of both *FOS* (−0.884 ± 0.319) and 18S rDNA (−0.801 ± 0.588) *FOS* is a component of the AP-1 transcription factor that regulates distinct cellular processes including cell proliferation, death, survival and differentiation [49] and under stress conditions regulation of ribosome biosynthesis is one of the cellular strategies to preserve homeostasis [50]. The large variation (standard deviation) associated with the gene expression data is of particular interest, especially in regards to *SPARC* and *NLC* mRNA levels. Considering that the levels of variation between technical replicates were almost absent, this shows that BPA induces general and non-directional de-regulation of gene expression in senescent cells. This is further supported by the transcription analysis of L1 related sequences after continuous BPA exposure (693 h, p19) showing a decrease in L1-5' (−0.549 ± 0.368) and an increase in L1-3' (1.115 ± 0.15) (Figure 3c).

Overall, the gene transcription results obtained on senescent HUVEC continuously exposed to BPA clearly show that this chemical affects the aging process. HUVEC constitute an *in vitro* model in the investigation of atherosclerosis pathogenesis [35], an intrinsically age-related disease in which vascular senescence play a critical role [23]. Relevantly, atherosclerosis has been related to both deficient endothelial cell turnover [51], as well as increased cell death [52]. Moreover, several epidemiologic studies indicate that circulating BPA is correlated with coronary and carotid atherosclerosis [20,21,22]. Overall, our results indicate that BPA may play a role in atherosclerosis induction by decreasing proliferation capacity in association with transcriptional de-regulation.

## 3. Experimental Section

### 3.1. Cell Cultures and Reagents

The effects of BPA were analyzed on HT29 and HUVEC representative of digestive and vascular tract tissues, respectively, which are exposed to BPA *in vivo*. HT29 human colon adenocarcinoma cell line was purchased from the European Collection of Cell Cultures (ECACC, London, UK) and was cultivated in 75 cm^2^ flasks with RPMI media containing GlutaMAX™ (#61870010, Invitrogen, Carlsbad, CA, USA), supplemented with 10% (*w*/*v*) fetal bovine serum (#16000-044, Invitrogen), 100 U/mL penicillin, 100 mg/mL streptomycin and 2 mM l-glutamine. Primary vascular endothelial (HUVEC) cells were kindly provided by Neo-vascularization Lab (IMM, Lisbon, Portugal), and cultivated in 75 cm^2^ flasks coated with 0.2% (*w*/*v*) gelatin. HUVEC were grown in EGM-2 media supplemented with the respective specific BulletKit (#CC-3156 and #CC-4176, LONZA, Basel, Switzerland) and with 5% (*v*/*v*) fetal bovine serum and 0.2% (*w*/*v*) Bovine Brain Extract (#CC-4098, LONZA). All cell cultures were maintained in a humidified 5% (*v*/*v*) CO_2_ in air atmosphere at 37 °C. For treatments and experiments, HUVEC were used between passages 3–6 (young HUVEC) and passages 11–19 (aging HUVEC). For aging cell passages were performed every 3–4 days independently of the confluence status and seeded at an identical density (3.2 × 10^3^ cel/cm^2^) for all conditions assayed.

### 3.2. BPA Treatments and Controls

After subculture procedure cells were stabilized for 24 h in standard growth medium before all experiments. BPA was freshly diluted in ethanol and added to the culture media to final concentrations of 10 ng/mL (44 nM) or 1 µg/mL (4.4 µM). As negative controls cells were grown in either standard culture media or in media supplemented with ethanol (vehicle) at a final concentration of 0.17 mM, corresponding to the vehicle added for both BPA concentrations assayed. For short exposure treatments, HT29, young (passages 4 and 6) and aging (passages 12) HUVEC were incubated in medium with BPA or control media for 24 or 72 h. For long and continuous BPA exposure, HUVEC were maintained in medium with or without BPA from passage 12 to passage 19. At each passage, cells were collected by trypsinization and cellular viability evaluated with trypan blue.

### 3.3. cDNA Isolation and Quantitative Real-Time Polymerase Chain Reaction (qRT-PCR)

Transcriptional analysis was performed by qRT-PCR for two distinct regions of the LINE1 retrolement (L1-3' and L1-5') and for osteonectin (*SPARC*), fibronectin (*FN1*), *p21*, *FOS*, *bcl-xL*, *NCL* and *18S rRNA* genes as well as control genes encoding *GAPDH* and *β-actin*, using the specific primers listed in Table 1.

Total RNA was extracted from trypsinized cells with the RNAqueous Kit (#AM1912, Invitrogen) following manufacturers’ instructions. After verifying concentration and integrity, 3 μg of total RNA was utilized for RNase free DNase digestion (RQ1 RNase free DNase, #M6101, Promega, Madison, WI, USA) and first strand cDNA synthesis was completed with random primers (DYNAmo cDNA syntesis Kit, #F-470L, Thermo Scientific, Waltham, MA, USA). The resulting cDNA was utilized for qRT-PCR with SsoFast Eva Green Supermix (#172-5201, BioRad, Hercules, CA, USA) utilizing the following conditions; 95 °C–3 min, 35 cycles (95 °C–30 s, 55 °C–30 s), 72 °C–5 min 40 °C. To ensure that genomic DNA was completely absent prior to cDNA synthesis, PCRs were performed with 18S primers and 250 ng of DNase digested RNA. Control PCRs were also performed for both primer combinations without template. Transcriptional analysis experiments were repeated at least three times and in each experiment at least three technical replicates per cell treatment/primer combination were performed. All comparisons of expression levels were performed on identical cDNA dilutions. After denaturation curves were observed to ensure correct amplification products, threshold cycles (*C*_t_) were equilibrated with mean GAPDH and β-actin to calculate ∆*C*_t_ (∆*C*_t_ = *C*_t_ of interest − mean (GAPDH–β-actin) *C*_t_) since no significant differences were detected between the two reference genes. Gene expression levels were analyzed by calculating ∆∆*C*_t_ (∆∆*C*_t_ = ∆*C*_t_ a − mean ∆*C*_t_ b, where medium a and b are being compared). Results are presented as log2 of the mean fold change (2^−∆∆*C*t^) ± standard deviation. Student’s *t* test was used for statistical analysis.

**Table 1:** Primers used for Quantitative Real-Time Polymerase Chain Reaction (qRT-PCR).

Sequence	Accession No.	Forward Primer (5'→3')	Reverse Primer (5'→3')
L1-5' [24]	M80340.1	GGCCAGTGTGTGTGCGCACCG	CCAGGTGTGGGATATAGTCTCGTGG
L1-3' [24]	M80340.1	CAGGAAGGGGAATATCACACTC	TGCGCTGCACCCACTAACTC
*SPARC*	NM_003118	CTGTGGGAGCTAATCCTG	GGGTGCTGGTCCAGCTGG
*FN1*	NM_002026	TGTGGTTGCCTTGCACGAT	GCTTGTGGGTGTGACCTGAGT
*p21*	NM_000389	CTGGAGACTCTCAGGGTCGAA	CCAGGACTGCAGGCTTCCT
*FOS*	NM_005252	AGGAGAATCCGAAGGGAAAG	CAAGGGAAGCCACAGACATC
*bcl-xL*	NM_001191	TTACCTGAATGACCACCTA	ATTTCCGACTGAAGAGTGA
*NCL*	NM_005381	CCTTCTGAGGACATTCCAAGACA	ACGGTATTGCCCTTGAAATGTT
*18SrRNA*	NR_003286	CATTCGAACGTCTGCCCTAT	CCTCCAATGGATCCTCGTTA
*GAPDH*	NM_002046	GAGTCAACGGATTTGGTCGTA	GCAGAGATGATGACCCTTTTG
*β-actin*	NM_001101	GGTCATCTTCTCGCGGTTGGCCTTGGGGT	CCCCAGGCACCAGGGCGTGAT

### 3.4. Protein Extraction and Western Blotting Analysis

For total protein lysate, cell pellets of trypsinized cultures were resuspended in 500 μL SDS buffer (0.125 M Tris–HCl, 10% (*v*/*v*) 2-mercaptoethanol, 2% (*w*/*v*) SDS and 10% (*w*/*v*) sucrose) [32] and sonicated on ice 2 × 15 s. After centrifugation (14,000× *g*), the supernatant was transferred to a fresh centrifuge tube and stored at −20 °C. Protein concentration was determined by the Bradford method (Protein assay, #500-0006, BioRad) and electrophoresis on polyacrylamide gels was performed using 50 µg of protein samples, transferred onto polyvinylidene difluoride membranes (PVDF) membranes, and stained by PonceauS reagent. The immunoblots were blocked with 3% (*w*/*v*) dry milk in PBST (0.05% (*v*/*v*) Tween 20, 137 mM NaCl, 1.5 mM KH_2_PO_4_, 8.1 mM Na_2_HPO_4_, 2.7 mM KCl) and incubated with primary antibodies: anti-trimethyl-histone H3 (Lys 4) (#ab8580, dilution 1:500; Abcam, London, UK) or anti-dimethyl-histone H3 (Lys 9) (#ab1220, dilution 1:2000; Abcam), and anti-α-tubulin (#T9026, dilution 1:2000; Sigma-Aldrich, St. Louis, MO, USA). Secondary peroxidase-conjugated anti-rabbit and anti-mouse antibodies (#32460 and #32430, respectively; Pierce Biotechnology, Rockford, IL, USA) were used (dilutions 1:1250). Detection was performed with SuperSignal West Femto Maximum sensitivity substrate kit (#34096, Thermo Scientific) and BioRad Chemidoc XRS. Two independent protein extractions for each treatment were performed and Western blots repeated for reproducibility.

### 3.5. Cell Viability Assay

Cell viability assessment was performed on 96-well dishes using CellTiter-Blue Viability Assay (#G8081, Promega) according to manufacturer’s instructions. After 4 h incubation fluorescence emission at 590 nm was measured using BioTek microplate reader Synergy HT. Cultivation and viability assay was performed simultaneously for all growth conditions. Statistic analysis was performed comparing treated cells with cells grown in standard medium. At least three independent experiments were performed with three replicates each per treatment. Student’s *t* test was used for statistical analysis.

### 3.6. Immunofluorescence

Cells were grown over glass coverslips coated with 0.2% (*w*/*v*) gelatin. After treatments, cells were fixed in 4% (*v*/*v*) paraformaldehyde for 10 min at room temperature and permeabilized with 0.25% (*v*/*v*) Triton X-100 for 15 min. Fixed cells were then incubated in 5% (*w*/*v*) BSA/PBS solution for 60 min and then incubated overnight at 4 °C with α-tubulin primary antibody (# ab7291, Abcam) diluted 1:200 in 1% (*w*/*v*) BSA/PBS. Detection was performed with conjugated anti-mouse-Cy3 IgG (#C2181, Sigma-Aldrich) secondary antibody diluted 1:200 in 1% (*w*/*v*) BSA/PBS for 60 min at 37 °C. Cells were then DAPI stained, and coverslips mounted on glass slides with antifade AF1 (Citifluor, London, UK). Immunofluorescence images were recorded with epifluorescence microscope Axioskop2 (Carl Zeiss, Oberkochen, Germany) equipped with a AxioCam MRc5 (Carl Zeiss) digital camera and superimposed with Adobe Photoshop 7.0 (Adobe Systems, San Jose, CA, USA) software.

## 4. Conclusions

For the past years, bisphenol A (BPA) has been implicated in the etiology of several human pathologies and its effects extensively studied. However, the current available information regarding BPA effects on aging processes is still scarce.

The present work demonstrates that BPA at low concentrations has the capacity to affect global transcription levels in both HT29 and HUVEC. Furthermore, we show for the first time that continuous exposure to BPA interferes with gene expression and severely decreases cellular viability in aging vascular endothelial cells. These results support the correlation between BPA and atherosclerosis related diseases reported in distinct epidemiological studies and increasing concerns regarding the adverse effects of BPA exposure on human health.
